# Early-Onset Parkinson's Disease Caused by PLA2G6 Compound Heterozygous Mutation, a Case Report and Literature Review

**DOI:** 10.3389/fneur.2019.00915

**Published:** 2019-08-21

**Authors:** Ting Shen, Jing Hu, Yasi Jiang, Shuai Zhao, Caixiu Lin, Xinzhen Yin, Yaping Yan, Jiali Pu, Hsin-Yi Lai, Baorong Zhang

**Affiliations:** ^1^Department of Neurology of the Second Affiliated Hospital, Zhejiang University School of Medicine, Zhejiang University, Hangzhou, China; ^2^Department of Neurology of the Second Affiliated Hospital, Interdisciplinary Institute of Neuroscience and Technology, Zhejiang University School of Medicine, Key Laboratory of Medical Neurobiology of Zhejiang Province, Zhejiang University, Hangzhou, China; ^3^Key Laboratory of Biomedical Engineering of Ministry of Education, Qiushi Academy for Advanced Studies, College of Biomedical Engineering and Instrument Science, Zhejiang University, Hangzhou, China

**Keywords:** Parkinson's disease, early-onset, PLA2G6, genetic analysis, neuroimaging

## Abstract

PLA2G6 has been certified as a causative gene in patients with autosomal recessive early-onset Parkinson's disease (EOPD). We reported an EOPD case caused by PLA2G6 gene mutation, and performed neurological examination, genetic analysis, and multimodal neuroimaging to describe this phenotype. A compound heterozygous mutation c.991G>T/c.1472+1G>A was detected in this patient. Heterozygous for the c.991G>T and c.1472+1G>A were separately detected in his parents. Pathogenicity of these two mutations were predicted according to the American college of medical genetics and genomics (ACMG) guideline. MRI assessment showed absence of bilateral “swallow tail sign” and cerebellar atrophy in this patient, while no obvious difference in brain iron accumulation between PLA2G6 mutant PD patient and healthy controls. Cerebellar abnormalities may be a marker for diagnosis and evaluation of PLA2G6 mutation Parkinsonism. However, the iron accumulation in PD may not be the result of PLA2G6 mutation.

## Introduction

Parkinson's disease (PD) is a chronic progressive neurodegenerative disorder, characterized by complex clinical symptoms including motor symptoms like resting tremor, bradykinesia, rigidity, gait abnormalities, and non-motor symptoms like hyposmia, dyssomnia, affective disturbance. Both genetic and environmental factors are believed to be involved in cause of PD (1). Parkin, PINK1, and DJ1 genes, had been identified to be responsible for autosomal recessive early-onset Parkinsonism with typical symptoms, while atypical parkinsonism caused by mutations in ATP13A2, FBXO7, DNAJC6, SYNJ1, and PLA2G6 genes displaying more complex phenotypes (2).

The PLA2G6 gene encodes calcium-independent phospholipase A2 beta enzyme (iPLA_2_β), which participates in cell membrane homeostasis, mitochondrial function, fatty acid oxidation, and calcium signaling (3, 4). PLA2G6 dysfunction has been proved as a pathogenic factor for PLA2G6-associated neurodegeneration (PLAN) (5), including infantile neuroaxonal dystrophy (INAD), neurodegeneration with brain iron accumulation (NBIA), and PLA2G6-related autosomal recessive dystonia-parkinsonism in its disease spectrum (6). Several mutations in PLA2G6 gene were reported to be associated with both atypical autosomal recessive parkinsonism (3, 7, 8), and sporadic early-onset PD (9, 10). PLA2G6 mutant parkinsonism cases showed alpha-synuclein pathology (11) in the substantia nigra and locus ceruleus, and one third of patients might be accompanied by iron accumulation (12).

In the present study, we reported an early-onset PD (EOPD) patient with compound heterozygous mutation in PLA2G6 gene, as well as compared with his mother and three health controls using neurological examination, genetic analysis and multimodal magnetic resonance imaging (MRI) to characterize the PLA2G6 mutation phenotype.

## Materials and Methods

### Subjects

This study was approved by the ethics committee of Second Affiliated Hospital of Zhejiang University School of Medicine. A patient with EOPD from a Chinese family was recruited from the outpatient clinic of the Department of Neurology, and four of his family members were also enrolled. In addition, we recruited three unrelated healthy controls at similar age of patient, without mutations of PLA2G6 gene. Clinical presentations including medical history, physical examination, and scale assessment as well as biological sample including peripheral blood were collected from all subjects.

### Genotyping

Genomic DNA was extracted from peripheral blood leukocytes using Blood Genomic Extraction Kit (Qiagen, Hilden, Germany). We had routinely screened several common pathogenic genes of early-onset parkinsonism including Parkin, PINK1, DJ1, ATP13A2, FBXO7, DNAJC6, SYNJ1 genes before, and no pathogenic mutations was detected in this case. We further amplified the exons and intron/exon boundaries of PLA2G6 gene (Supplementary Table 1) by polymerase chain reaction (PCR) and then directly sequenced using an ABI 3730 XL genetic analyzer (Applied Biosystems, Foster City, USA). Alignment and analysis of the sequencing results was carried out with DNAStar (DNAStar, In Madison, WI). Multiplex ligation-dependent probe amplification (MLPA) assay (SALSA MLPA kit P120-B2, MRC-Holland) was used to detect exon deletion and duplication of PLA2G6.

### *In silico* Prediction Analysis

To predict the potential pathogenicity of genetic variants, *in silico* prediction analysis was performed according to the American college of medical genetics and genomics (ACMG) guideline (13). Multiple sequence alignment of PLA2G6 protein sequences from different species was performed by ClustalX program to test the gene evolutionary conservation. Three *in silico* algorithms, including Mutation Taster (http://www.mutationtaster.org) (14), PolyPhen-2 (http://genetics.bwh.harvard.edu/pph2/) (15), and Mutation assessor (http://mutationassessor.org/r3/), were used to predict the effect of mutations on PLA2G6 function. Possible changes of the splice sites were predicted using NNSplice program encoded in Mutation Taster program. 3-D protein structures of both wild and variant type PLA2G6 protein were predicted using an *ab initio* modeling server, I-TASSER program (https://zhanglab.ccmb.med.umich.edu/I-TASSER-MR/) (16), which were then viewed and edited by the molecular visualization system PyMOL (PyMOL Molecular Graphics System, Version 1.5, Schrödinger, LLC). The missense variant was classified as “Benign” (class 1), “Likely benign” (class 2), “Uncertain significance” (class 3), “Likely pathogenic” (class 4), and “Pathogenic” (class 5) using the automated pathogenicity tool, InterVar software (http://wintervar.wglab.org/, only non-synonymous variants have automated ACMG interpretation so far) (17), which can generate the preliminary interpretation according to the ACMG guideline. The splice site variant was evaluated manually based on the ACMG guideline.

### Neuroimaging Analysis

Multimodal MR images were acquired by a 7.0T MRI research system (Magnetom, Siemens Healthcare, Erlangen, Germany) with prototype sequences, including magnetization prepared two rapid gradient echoes (MP2RAGE) sequence (voxel size: 0.7 × 0.7 × 0.7 mm, TR = 5,000 ms, TI1/TI2 = 900/2,750 ms, TE = 2.3 ms, α1/α2 = 5°/3°), gradient echo (GRE) based susceptibility weighted imaging (SWI) sequence (voxel size: 0.25 × 0.25 × 1.5 mm, TR = 27 ms, TE = 15 ms) and multi-band echo planar diffusion tensor image (DTI) sequence (voxel size: 1.5 × 1.5 × 1.5 mm, TR = 6,000 ms, TE = 87.4 ms). The DTI data was processed using FSL, Diffusion Toolkit, and TrackVis software.

## Results

### Clinical Presentation

**Figure 1:**
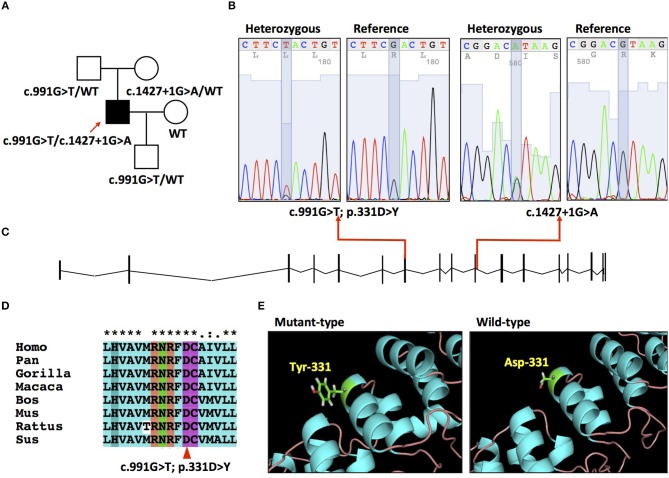
**(A)** Pedigree of the family. **(B)** Electropherograms of the mutation c.991G>T and c.1427 + 1G>A. **(C)** Schematic of PLA2G6 gene (NM_003560.2) and locations of mutations. **(D)** Conservation of amino acid residue across different species in PLA2G6 gene. **(E)** The 3-D structures of wild type and mutant-type proteins.

A 33-year-old male patient developed gait abnormality and posture balance disturbance at age of 30 years. In the following 3 years, his symptoms progressed and developed increasing rigidity, bradykinesia, propulsive gait, obvious cerebellar signs like ataxia, imbalance, low limb fatigue, and frequent falls. Cognitive decline was also observed. He was treated with levodopa (300 mg/day), amantadine (200 mg/day), piribedil (50 mg/day), benzhexol (2 mg/day), showing good response but appeared levodopa-induced dyskinesia. Certain rating scales were performed to evaluate his clinical condition after 12 h withdrawal of anti-parkinsonism medication during “OFF” stage, (MDS-UPDRS I, II, III, IV, scores: 13, 27, 60, 11; Hoehn-Yahr scale: 4; MoCA: 15; HAMA: 15; HAMD: 15). Then the patient took his daily dose of anti-Parkinson's disease drugs, which took him almost 1 h to show effectiveness. The clinical condition during “ON” stage was also accessed (MDS-UPDRS III, score: 34; Hoehn-Yahr scale: 3), indicating 43.3% improvement of motor symptoms and accompanied by mild dyskinesia. There was no history of parkinsonism and related disease in his previous generations, and his parents were not consanguineous marriage (Figure 1A). Both parents showed no neurological and neuropsychological manifestations.

### Genetic Analysis

A compound heterozygous mutation c.991G>T/c.1472+1G>A was detected in the patient (Figure 1B). MPLA analysis ruled out the presence of exon deletion or duplication of PLA2G6. His father and mother resulted heterozygous for the c.991G>T and c.1472+1G>A separately. His wife didn't carry PLA2G6 mutation. His son was detected heterozygous for the c.991G>T mutation. The c.991G>T mutation is located in exon 7 of the NM_003560 transcript (Figure 1C), which causes a p.331D>Y transition and leads to aspartic acid-tyrosine change in the seventh ankyrin repeats (ranging from amino acid 150 to 382) (Figure 1E). This mutation is reported as “pathogenic” in ClinVar database and presents in heterozygous status in ExAC and 1,000 Genomes database with fairly low allele frequency (0.00005 and 0.0002). The number 331 aspartic acid is highly conserved across different species (Figure 1D). It is predicted to be “disease causing” by Mutation Taster, “medium impact” by Mutation assessor, “possibly damaging” by Polyphen2. The automated clinical interpretation of the c.991G>T mutation generated by the InterVar software is “Pathogenic” (class 5).

The c.1472+1G>A mutation is located in the junction between exon 10 and intron 10, acts as a splice donor variant affecting its splicing (Figure 1C). It is reported as “likely pathogenic” in ClinVar database and presents in heterozygous status in ExAC database with a very low allele frequency (0.000008). This mutation is predicted to be “disease causing” by Mutation Taster and “likely to disturb normal splicing” by NNSPLICE. We classified this splice site mutation into “Uncertain significance” (class 3), according to the ACMG guideline.

### Neuroimaging Assessment

**Figure 2:**
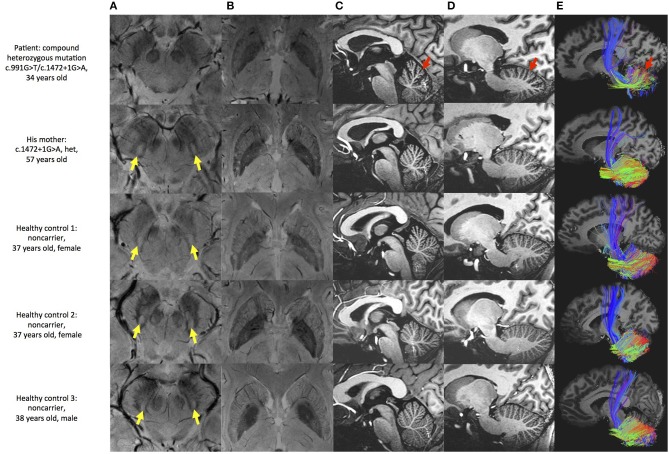
Brain MRI examination of the patient and controls. **(A)** SWI image shows substantia nigra and red nucleus. Hyperintensity is found in substantia nigra of patient's mother and healthy controls (yellow arrow), but not in this patient. **(B)** SWI image shows basal ganglia. Hypointensity can be seen in globus pallidus and margin of putamen in all subjects. **(C,D)** MP2RAGE sagittal image shows deepened cerebellar fissures in this patient (red arrow). **(E)** Fiber tracking shows sparser tract in the patient (red arrow).

Multimodal MRI was performed in the patient, patient's mother and three healthy controls. SWI images showed that the patient presented the absence of bilateral nigrosome-1 hyperintensity, also known as “swallow tail sign,” while it was presented in patient's mother and healthy controls (Figure 2A). The results showed atrophy and iron deposition of substantia nigra in the patient. Hypointense signals in bilateral globus pallidus and margin of putamen were found in all subjects (Figure 2B). MP2RAGE structural images also showed cerebellar atrophy in the patient as compared with his mother and three age-matched healthy controls (Figures 2C,D). The cerebellar white matter fiber tracts from this patient was sparser than controls, providing more evidence of cerebellar atrophy (Figure 2E).

## Discussion

In the current study, we reported a case of early-onset PD caused by a compound heterozygous mutation of c.991G>T/c.1472+1G>A. His initial symptoms were gait and posture disturbance with relatively early-onset and quick progression, developed rigidity, dyskinesia, and cognitive decline, basically conformed to PLA2G6 mutation-induced phenotype. The PLA2G6 gene encodes iPLA_2_β enzyme, participates in various kinds of cellular functions including phospholipid metabolism, membrane homeostasis, mitochondrial function, calcium signaling, apoptosis, and inflammatory response (18), which would be disturbed by gene mutations. The c.991G>T mutation had been demonstrated that led to degeneration of dopaminergic neurons in substantia nigra compact by causing mitochondrial dysfunction, elevated endoplasmic reticulum stress, and transcriptional abnormality (4), conforming its pathogenicity. The c.1472+1G>A was considered as a “likely pathogenic” mutation, which had been found in INDA patient recorded in ClinVar database (https://preview.ncbi.nlm.nih.gov/clinvar/variation/437465), but hadn't been reported in PLA2G6-related parkinsonism. Together, this compound heterozygous mutation was first detected in parkinsonism and could be regarded as a causative factor for this phenotype.

On review of previous literatures, we summarized PLA2G6 homozygous and compound heterozygous mutations reported in the PLA2G6-related parkinsonism (Table 1). These mutations were discovered in various races, classified into “Uncertain significance” (Class 3), “Likely pathogenic” (class 4) or “Pathogenic” (class 5) according to the ACMG guideline. Most cases initiated symptoms in their childhood or early adult, the onset age varies from 8 to 36. Although there is clinical heterogeneity, patients commonly showed obvious dystonia, ataxia, good Levodopa response, Levodopa-induced dyskinesia, and cognitive decline. Our case also conforms to the common phenotype.

**Table 1:** Homozygous and compound heterozygous mutations of patients with PLA2G6-related Parkinsonism.

**Mutation**	**Protein change change**	**ACMG class^**a**^**	**Reference**
c.4C>A/Del Ex 3	p.Gln2Lys/p.Leu71_Ser142del	4	(19)
c.109C>T/c.1078-3C>A	p.Arg37X/–	5	(20)
c.109C>T/c.2321G>T	p.Arg37X/p.Ser774Iso	5	(21)
c.216C>A/c.1904G>A	p.Phe72Leu/p.Arg635Gln	4	(6)
c.610-1G>T/c.1627C>T	–/p.Arg543Cys	3	(11)
c.758G>T/c.2341G>A	p.Gly253Val/p.Ala781Thr	3	(21)
c.991G>T	p.Asp331Tyr	5	(22–24)
c.991G>T/c.1077G>A	p.Asp331Tyr/p.Met358IlefsX	5	(23)
c.1039G>A/c.1670C>T	p.Gly347Arg/p.Ser557Leu	3	(25)
c.1354C>T/c.1904G>A	p.Gln452X/p.Arg635Gln	5	(6)
c.1495G>A	p.Ala499Thr	3	(26)
c.1547C>T	p.Ala516Val	3	(27)
c.1715C>T	p.Thr572Ile	3	(20)
c.1791delC	p.His597fx69	5	(10)
c.1894C>T	p.Arg632Trp	3	(28)
c.1966C>G	p.Leu656Val	3	(10)
c.2077C>G	p.Leu693Val	3	(10)
c.2215G>C	p.Asp739His	4	(29)
c.2222G>A	p.Arg741Gln	4	(8, 12, 30, 31)
c.2239C>T	p.Arg747Trp	4	(30, 32, 33)
c.2339A>G	p.Asn780Ser	3	(34)
c.2341G>A	p.Ala781Thr	3	(34)

a *ACMG classification: 5, pathogenic; 4, likely pathogenic; 3, uncertain significance; 2, likely benign; and 1, benign*.

Iron accumulation generates toxic α-synuclein or tau aggregates bring damage to the dopaminergic neurons, which contribute to the pathogenesis of PD (35). PLAN cases are often accompanied by iron accumulation (5), while one of those types, the PLA2G6 mutant Parkinsonism cases are observed with or without iron accumulation. We found no difference in hypointense signal in bilateral basal ganglia between patient and controls indicated that the PLA2G6 mutant PD patient had no obvious iron deposit. Guo et al. (36) found that decreased iPLA2β activity might cause decreased uptake activity and increased iron storage activity, but didn't lead to iron accumulation. We assume that PLA2G6 mutation may not be the main cause of iron accumulation of PD.

Cerebellar atrophy is an early sign commonly seen in patients with PLAN, such as infantile neuroaxonal dystrophy (INAD), neurodegeneration with brain iron accumulation (NBIA) and PARK14-linked autosomal recessive early-onset dystonia-parkinsonism (12, 37), and also observed in this case. Zhao et al. found that the absence of PLA2G6 might cause neuroinflammation and loss of Purkinje cell, and eventually leaded to cerebellar atrophy. Darling et al. (37) performed quantitative assessment of cerebellar atrophy, and found it correlated with the severity of disease phenotype. We suggested that the cerebellar abnormalities may be an important feature of PLAN.

In conclusion, we identified a pathogenic compound heterozygous mutation of PLA2G6 in an EOPD patient, exhibiting PLA2G6 mutation-induced phenotype. Results of multimodal MRI showed cerebellar atrophy while normal iron level in basal ganglia indicated that cerebellar abnormalities might be a marker for diagnosis and evaluation of PLA2G6 mutation Parkinsonism. Furthermore, we demonstrated that PLA2G6 mutation might not result in iron accumulation in PD.

## Data Availability

All datasets generated for this study are included in the manuscript and the Supplementary Files.

## Ethics Statement

All adult subjects gave written informed consent for the publication of this case report. And written informed consent of underage subject was obtained from the parents.

## Author Contributions

TS, H-YL, and BZ designed this study. XY, YY, and JP were responsible for diagnosis and clinical evaluation. JH, CL, and SZ performed genetic analysis. TS, YJ, and H-YL collected and analyzed MRI data. TS wrote the manuscript. H-YL and BZ contributed to the revision of the manuscript.

### Conflict of Interest Statement

The authors declare that the research was conducted in the absence of any commercial or financial relationships that could be construed as a potential conflict of interest.

## References

[1] Poewe W Seppi K Tanner CM Halliday GM Brundin P Volkmann J. Parkinson disease. Nat Rev Dis Primers. (2017) 3:17013. 10.1038/Nrdp.2017.13 28332488

[2] Sheerin UM Houlden H Wood NW. Advances in the genetics of Parkinson's disease: a guide for the clinician. Mov Disord Clin Pract. (2014) 1:3–13. 10.1002/mdc3.12000 30363913 PMC6183020

[3] Ferese R Scala S Biagioni F Giardina E Zampatti S Modugno N. Heterozygous PLA2G6 mutation leads to iron accumulation within basal Ganglia and Parkinson's disease. Front Neurol. (2018) 9:536. 10.3389/fneur.2018.00536 30042723 PMC6048271

[4] Chiu CC Lu CS Weng YH Chen YL Huang YZ Chen RS. PARK14 (D331Y) PLA2G6 causes early-onset degeneration of substantia nigra dopaminergic neurons by inducing mitochondrial dysfunction, ER stress, mitophagy impairment and transcriptional dysregulation in a knockin mouse model. Mol Neurobiol. (2018) 56:3835–53. 10.1007/s12035-018-1118-5 30088174

[5] Morgan NV Westaway SK Morton JE Gregory A Gissen P Sonek S. PLA2G6, encoding a phospholipase A2, is mutated in neurodegenerative disorders with high brain iron. Nat Genet. (2006) 38:752–4. 10.1038/ng1826 16783378 PMC2117328

[6] Yoshino H Tomiyama H Tachibana N Ogaki K Li Y Funayama M. Phenotypic spectrum of patients with PLA2G6 mutation and PARK14-linked parkinsonism. Neurology. (2010) 75:1356–61. 10.1212/WNL.0b013e3181f73649 20938027

[7] Paisan-Ruiz C Guevara R Federoff M Hanagasi H Sina F Elahi E. Early-onset L-dopa-responsive parkinsonism with pyramidal signs due to ATP13A2, PLA2G6, FBXO7 and spatacsin mutations. Mov Disord. (2010) 25:1791–800. 10.1002/mds.23221 20669327 PMC6005705

[8] Bohlega SA Al-Mubarak BR Alyemni EA Abouelhoda M Monies D Mustafa AE. Al Tassan: clinical heterogeneity of PLA2G6-related parkinsonism: analysis of two Saudi families. BMC Res Notes. (2016) 9:295. 10.1186/s13104-016-2102-7 27268037 PMC4897907

[9] Tian JY Tang BS Shi CH Lv ZY Li K Yu RL Shen L Yan XX Guo JF. Analysis of PLA2G6 gene mutation in sporadic early-onset parkinsonism patients from Chinese population. Neurosci Lett. (2012) 514:156–8. 10.1016/j.neulet.2012.02.078 22406380

[10] Gui YX Xu ZP Wen L Liu HM Zhao JJ Hu XY. Four novel rare mutations of PLA2G6 in Chinese population with Parkinson's disease. Parkinsonism Relat Disord. (2013) 19:21–6. 10.1016/j.parkreldis.2012.07.016 23182313

[11] Klein C Lochte T Delamonte SM Braenne I Hicks AA Zschiedrich-Jansen K. PLA2G6 mutations and Parkinsonism: long-term follow-up of clinical features and neuropathology. Mov Disord. (2016) 31:1927–9. 10.1002/mds.26814 27709683

[12] Karkheiran S Shahidi GA Walker RH Paisan-Ruiz C. PLA2G6-associated dystonia-Parkinsonism: case report and literature review. Tremor Other Hyperkinet Mov. (2015) 5:317. 10.7916/D84Q7T4W 26196026 PMC4503963

[13] Richards S Aziz N Bale S Bick D Das S Gastier-Foster J. Standards and guidelines for the interpretation of sequence variants: a joint consensus recommendation of the American College of Medical Genetics and Genomics and the Association for Molecular Pathology. Genet Med. (2015) 17:405–24. 10.1038/gim.2015.30 25741868 PMC4544753

[14] Schwarz JM Rodelsperger C Schuelke M Seelow D. MutationTaster evaluates disease-causing potential of sequence alterations. Nat Methods. (2010) 7:575–6. 10.1038/nmeth0810-575 20676075

[15] Adzhubei IA Schmidt S Peshkin L Ramensky VE Gerasimova A Bork P. A method and server for predicting damaging missense mutations. Nat Methods. (2010) 7:248–9. 10.1038/nmeth0410-248 20354512 PMC2855889

[16] Roy A Kucukural A Zhang Y. I-TASSER: a unified platform for automated protein structure and function prediction. Nat Protocols. (2010) 5:725–38. 10.1038/nprot.2010.5 20360767 PMC2849174

[17] Li Q Wang K. InterVar: clinical interpretation of genetic variants by the 2015 ACMG-AMP guidelines. Am J Hum Genet. (2017) 100:267–80. 10.1016/j.ajhg.2017.01.004 28132688 PMC5294755

[18] Kinghorn KJ Castillo-Quan JI Bartolome F Angelova PR Li L Pope S. Loss of PLA2G6 leads to elevated mitochondrial lipid peroxidation and mitochondrial dysfunction. Brain. (2015) 138(Pt 7):1801–16. 10.1093/brain/awv132 26001724 PMC4559908

[19] Bower MA Bushara K Dempsey MA Das S Tuite PJ. Novel mutations in siblings with later-onset PLA2G6-associated neurodegeneration (PLAN). Mov Disord. (2011) 26:1768–9 10.1002/mds.23617 21520282

[20] Paisan-Ruiz C Li A Schneider SA Holton JL Johnson R Kidd D. Widespread Lewy body and tau accumulation in childhood and adult onset dystonia-parkinsonism cases with PLA2G6 mutations. Neurobiol Aging. (2012) 33:814–23. 10.1016/j.neurobiolaging.2010.05.009 20619503 PMC3657696

[21] Wirth T Weibel S Montaut S Bigaut K Rudolf G Chelly J. Severe early -onset impulsive compulsive behavior and psychosis in PLA2G6-related juvenile Parkinson's disease. Parkinsonism Relat Disord. (2017) 41:127–9. 10.1016/j.parkreldis.2017.05.014 28549837

[22] Shi CH Tang BS Wang L Lv ZY Wang J Luo LZ. PLA2G6 gene mutation in autosomal recessive early-onset parkinsonism in a Chinese cohort. Neurology. (2011) 77:75–81. 10.1212/Wnl.0b013e318221acd3 21700586

[23] Lu CS Lai SC Wu RM Weng YH Huang CL Chen RS. PLA2G6 mutations in PARK14-linked young-onset parkinsonism and sporadic Parkinson's disease. Am J Med Genet B. (2012) 159B:183–91. 10.1002/ajmg.b.32012 22213678

[24] Xie F Cen ZD Ouyang ZY Wu S Xiao JF Luo W. Homozygous p.D331Y mutation in PLA2G6 in two patients with pure autosomal-recessive early-onset parkinsonism: further evidence of a fourth phenotype of PLA2G6-associated neurodegeneration. Parkinsonism Relat Disord. (2015) 21:420–2. 10.1016/j.parkreldis.2015.01.012 25660576

[25] Kim YJ Lyoo CH Hong S Kim NY Lee MS. Neuroimaging studies and whole exome sequencing of PLA2G6-associated neurodegeneration in a family with intrafamilial phenotypic heterogeneity. Parkinsonism Relat Disord. (2015) 21:402–6. 10.1016/j.parkreldis.2015.01.010 25634434

[26] Yamashita C Funayama M Li Y Yoshino H Yamada H Seino Y. Mutation screening of PLA2G6 in Japanese patients with early onset dystonia-parkinsonism. J Neural Transm. (2017) 124:431–5. 10.1007/s00702-016-1658-7 27942883

[27] Malaguti MC Melzi V Di Giacopo R Monfrini E Di Biase E Franco G. A novel homozygous PLA2G6 mutation causes dystonia-parkinsonism. Parkinsonism Relat Disord. (2015) 21:337–9. 10.1016/j.parkreldis.2015.01.001 25601130

[28] Sina F Shojaee S Elahi E Paisan-Ruiz C. R632W mutation in PLA2G6 segregates with dystonia-parkinsonism in a consanguineous Iranian family. Euro J Neurol. (2009) 16:101–4. 10.1111/j.1468-1331.2008.02356.x 19087156

[29] Kamel WA Al-Hashel JY Abdulsalam AJ Damier P Al-Mejalhem AY. PLA2G6-related parkinsonism presenting as adolescent behavior. Acta Neurol Belg. (2018). [Epub ahead of print]. 10.1007/s13760-018-1003-z. 30120687

[30] Paisan-Ruiz C Bhatia KP Li A Hernandez D Davis M Wood NW. Characterization of PLA2G6 as a locus for dystonia-parkinsonism. Ann Neurol. (2009) 65:19–23. 10.1002/ana.21415 18570303 PMC9016626

[31] Virmani T Thenganatt MA Goldman JS Kubisch C Greene PE Alcalay RN. Oculogyric crises induced by levodopa in PLA2G6 parkinsonism-dystonia. Parkinsonism Relat Disord. (2014) 20:245–7. 10.1016/j.parkreldis.2013.10.016 24182522

[32] Erro R Balint B Kurian MA Brugger F Picillo M Barone P. Early ataxia and subsequent parkinsonism: PLA2G6 mutations cause a continuum rather than three discrete phenotypes. Mov Disord Clin Pract. (2017) 4:125–8. 10.1002/mdc3.12319 30868093 PMC6407056

[33] Giri A Guven G Hanagasi H Hauser AK Erginul-Unaltuna N Bilgic B. PLA2G6 mutations related to distinct phenotypes: a new case with early-onset parkinsonism. Tremor Other Hyperkinet Mov. (2016) 6:363. 10.7916/D81G0M12 27127721 PMC4811020

[34] Kauther KM Hoft C Rissling I Oertel WH Moller JC. The PLA2G6 gene in early-onset Parkinson's disease. Mov Disord. (2011) 26:2415–7. 10.1002/mds.23851 21812034

[35] Lee JH Lee MS. Brain iron accumulation in atypical parkinsonian syndromes: *in vivo* MRI evidences for distinctive patterns. Front Neurol. (2019) 10:74 10.3389/fneur.2019.00074 30809185 PMC6379317

[36] Guo YP Tang BS Liu HL Huang JJ Xu Q Sun QY. Impaired iPLA2beta activity affects iron uptake and storage without iron accumulation: an *in vitro* study excluding decreased iPLA2beta activity as the cause of iron deposition in PLAN. Brain Res. (2019) 1712:25–33. 10.1016/j.brainres.2019.01.036 30707893

[37] Darling A Aguilera-Albesa S Tello CA Serrano M Tomas M Camino-Leon R. PLA2G6-associated neurodegeneration: new insights into brain abnormalities and disease progression. Parkinsonism Relat Disord. (2018) 61:179–86. 10.1016/j.parkreldis.2018.10.013 30340910

