# Supplementary material for: Early-Onset Parkinson's Disease Caused by PLA2G6 Compound Heterozygous Mutation, a Case Report and Literature Review
Source: Front Neurol. 2019 Aug 21;10:915. doi: 10.3389/fneur.2019.00915 (PMC6712964; doi:10.3389/fneur.2019.00915)
Supplement: Supplementary file 1 [file Table_1.docx]

Supplementary Material

**Supplementary Table 1.** Primer sequences for PLA2G6 mutation screening

| Exons | PCR primers | Exons | PCR primers |
| --- | --- | --- | --- |
| Exon1 | F:5’>GGGGGACAGCCTTCTAGGTA<3’  R:5’>ACTGATGGGCCAGAAGTGTG<3’ | Exon9 | F:5’>CTGGGATCAGGACGAGGGAT<3’ <3’  R:5’>CCCAGCATTAATGAACGAGCG<3’ <3’ |
| Exon2 | F:5’>GCAGCCTGGGGACCTTC<3’  R:5’>ACTATGGAGGGGAACCGAGG<3’ | Exon10 | F:5’>TAGGACAGAAGTTCCTCGGGT<3’  R:5’>AAATGAAGTGTGCAGGCGAG<3’ |
| Exon3 | F:5’>AGTCCGAGTTTCCGAGTGC<3’  R:5’>AGCAAAGAGACTGAGGACGTG<3’ | Exon11 | F:5’>TTAGGCCTCGGTAAACCCG<3’  R:5’>GTCATTTGAAAGGCCAGTGGG<3’ |
| Exon4 | F:5’>AGAATCATTCCCACCTGGACCC<3’  R:5’>AGATCTATGGTGGATACTGCTTGC<3’ | Exon12 | F:5’>CCTATCCCGAACAGAGGTTGG<3’  R:5’>CTCTGGCTAGTTCGTCCTGG<3’ |
| Exon5 | F:5’>TTCCCACTCTGTCATACTGCTTC<3’  R:5’>ATCCCAGCTCTTCATGGACTT<3’ | Exon13 | F:5’>GTCCCTGGAAAGTCCTCAGC<3’  R:5’>GGTCCCTAGCATGGTTTGCT<3’ |
| Exon6 | F:5’>CCCAGTACCTGTAGGCCTCT<3’  R:5’>AAGGGAAGCAGGATGCTCAC<3’ | Exon14 | F:5’>ATGCTCTTGCCACCAGAGAC<3’  R:5’>GGATCCGCAGCTAAAAAGCG<3’ |
| Exon7 | F:5’>GCCTGGGTTCTTACACCCTC<3’  R:5’>ACGGGAGAGGATGCTGGTAT<3’ | Exon15/16 | F:5’>TCAGCCTGACTCGAAAGAGC<3’  R:5’>GGCTCTAGACTTTCCCAGCC<3’ |
| Exon8 | F:5’>TGGCTGGCTCATTAGTCCCT<3’  R:5’>CCTAGAGGCTGACAACTCCG<3’ |  |  |
